# Multiphasic Prehabilitation Across the Cancer Continuum: A Narrative Review and Conceptual Framework

**DOI:** 10.3389/fonc.2020.598425

**Published:** 2021-01-11

**Authors:** Daniel Santa Mina, Stefanus J. van Rooijen, Enrico M. Minnella, Shabbir M. H. Alibhai, Priya Brahmbhatt, Susanne O. Dalton, Chelsia Gillis, Michael P. W. Grocott, Doris Howell, Ian M. Randall, Catherine M. Sabiston, Julie K. Silver, Gerrit Slooter, Malcolm West, Sandy Jack, Franco Carli

**Affiliations:** ^1^ Faculty of Kinesiology and Physical Education, University of Toronto, Toronto, ON, Canada; ^2^ Department of Anesthesia and Pain Management, University Health Network, Toronto, ON, Canada; ^3^ Faculty of Medicine, University of Toronto, Toronto, ON, Canada; ^4^ Department of Surgical Oncology, Máxima Medical Center, Veldhoven, Netherlands; ^5^ Department of Anesthesia, McGill University Health Center, Montreal, QC, Canada; ^6^ Department of Anesthesia and Intensive Care, IRCCS San Raffaele Scientific Institute, Milano, Italy; ^7^ Danish Cancer Society Research Center, Copenhagen, Denmark; ^8^ Department of Clinical Oncology & Palliative Care, Zealand University Hospital, Næstved, Denmark; ^9^ Cumming School of Medicine, University of Calgary, Calgary, AB, Canada; ^10^ Faculty of Medicine, University of Southampton, Southampton, United Kingdom; ^11^ Acute Perioperative and Critical Care Theme, NIHR Southampton Biomedical Research Centre, University Hospital Southampton NHS Trust, University of Southampton, Southampton, United Kingdom; ^12^ Faculty of Nursing, University of Toronto, Toronto, ON, Canada; ^13^ Department of Physical Medicine and Rehabilitation, Harvard Medical School, Boston, MA, United States; ^14^ NIHR Biomedical Research Centre, University Hospital Southampton NHS Trusts, Southampton, United Kingdom

**Keywords:** cancer, survivorship, prehabilitation, rehabilitation, oncology, continuum of care, conceptual framework, enhanced recovery after surgery

## Abstract

The field of cancer survivorship has significantly advanced person-centered care throughout the cancer continuum. Within cancer survivorship, the last decade has seen remarkable growth in the investigation of prehabilitation comprising pre-treatment interventions to prevent or attenuate the burden of oncologic therapies. While the majority of evidence remains in the surgical setting, prehabilitation is being adapted to target modifiable risk factors that predict poor treatment outcomes in patients receiving other systemic and localized anti-tumor treatments. Here, we propose a multiphasic approach for prehabilitation across the cancer continuum, as a conceptual framework, to encompass the variability in cancer treatment experiences while adopting the most inclusive definition of the cancer survivor.

## Introduction

For more than thirty years, cancer survivorship has grown to become a well-established and internationally endorsed component of gold-standard, person-centered care that starts at diagnosis and continues to end of life. The seminal report on survivorship by the Institute of Medicine and the National Research Council, entitled “From Cancer Patient to Survivor: Lost in Transition” recently celebrated a decade’s worth of influence through its articulation of ten recommendations to improve oncology care (1). These recommendations specifically focus on the “period following first diagnosis *and* treatment and prior to the development of a recurrence of the initial cancer or death”, in response to insufficient attention to patients’ needs during this time. With remarkable progress in this field, pause for reflection on the application of survivorship principles at the core of these recommendations (*e.g.*, strategies to “identify and manage late effects of cancer and its treatment”) (2) is warranted, particularly, how these principles apply to the periods *between* diagnosis (*i.e.*, primary, recurrence, and second primary) and treatment(s).

Cancer rehabilitation programs aim to help a person maximize physical, social, psychological, and vocational functioning within the limits imposed by cancer and its treatment (3) and are often the crux of cancer survivorship services. Because the field of cancer rehabilitation predates survivorship terminology, its integration (although still a work in progress) reflects its medical origins in impairment-driven care. While representing a marked advancement in oncology, contemporary cancer rehabilitation has largely been reactive to treatment sequelae rather than proactive in preventing or attenuating anticipated consequences of common treatments. The ‘future’ of cancer rehabilitation in 1974 highlighted approaches to prevent or minimize disability that could be reasonably predicted; however, only recently have ‘rehabilitation’ models been proposed in which services are initiated at the time of diagnosis and continued throughout the continuum of treatment (4, 5). The focus of recent interventions on building resilience *prior to* treatment through conditioning and medical optimization is commonly referred to as *pre*habilitation.

Cancer prehabilitation is defined as “a process on the continuum of care that occurs between the time of cancer diagnosis and the beginning of acute treatment, includes physical and psychological assessments that establish a baseline functional level, identifies impairments, and provides targeted interventions that improve a person’s health to prevent or reduce the incidence and the severity of current and future impairments” (6). Prehabilitation is not oncology-specific, but is a growing field unto itself that has historically been applied to surgery, where preoperative physiological and psychosocial health are well-established predictors of peri- and postoperative outcomes (7, 8). Systematic reviews of prehabilitation in surgical oncology provide encouraging findings such as improved functional capacity, maintenance of lean mass, length of hospital stay, surgical complication rates, and health-related quality of life (HRQoL); however, methodological limitations have led to cautious interpretation (9–13).

**Figure 1:**
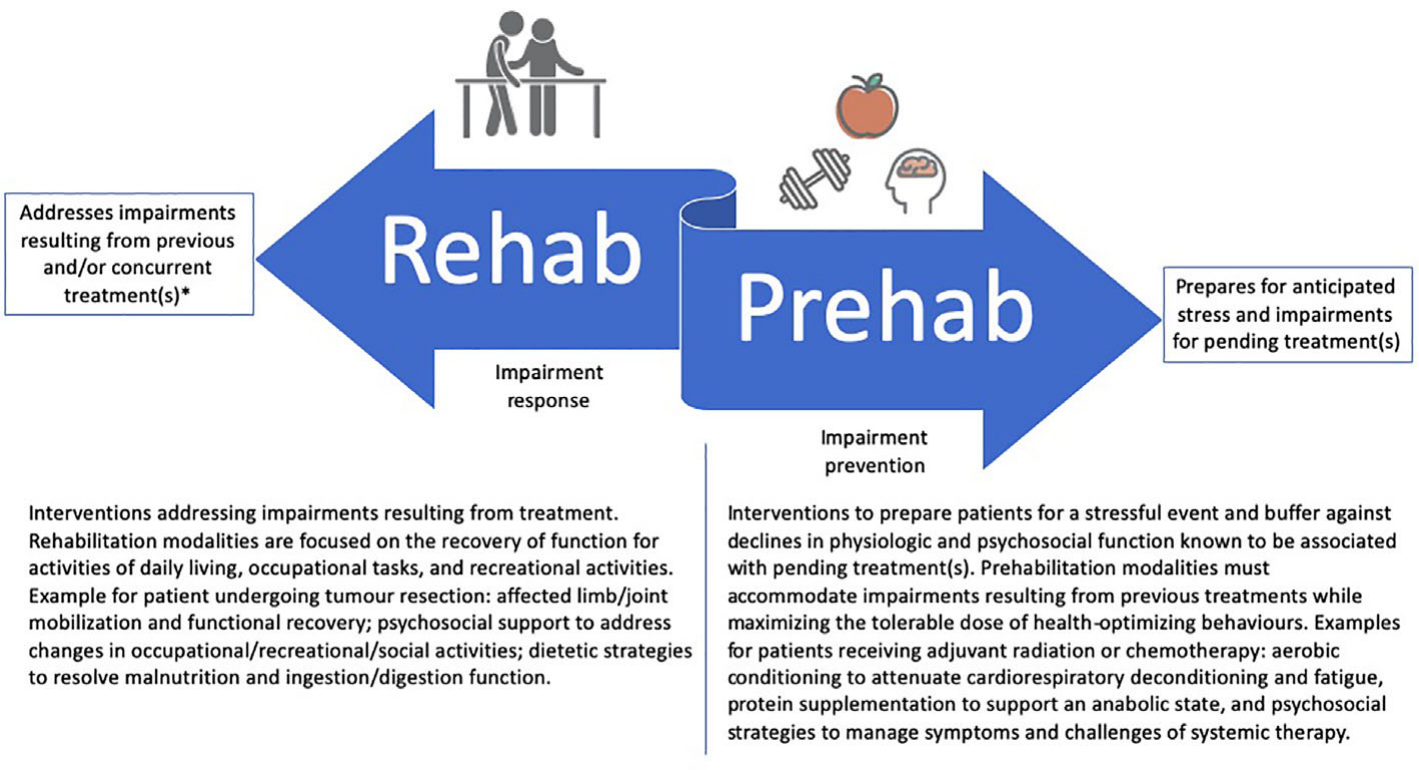
Example of coordinated rehabilitation and prehabilitation between two treatments.

The rapid growth of cancer prehabilitation research over the past decade has contributed to a push for clinical implementation within perioperative care models (14, 15) despite gaps in foundational prehabilitation frameworks that may limit its impact in practice. First, while prehabilitation models have nearly exclusively focused on the period between diagnosis and surgery, cancer is often treated with multiple lines of therapy, each with unique treatment-related sequelae and challenges to completion. Accordingly, multiple phases of prehabilitation may be needed to prepare for consecutive treatments and their unique anticipated adverse effects. Whereas referral or invitation to prehabilitation may currently reside with perioperative care physicians (*e.g.*, anesthetists and surgeons), extending prehabilitation to neoadjuvant and adjuvant treatments may offer opportunities for other physicians (*e.g.*, medical or radiation oncologists) to direct their patients to prehabilitation. Second, while prehabilitation may become an integral part of survivorship care, it does not intend to replace post-treatment rehabilitation, but rather, aims to complement it (**Figure 1**). For example, prehabilitation may include the education on early ambulation after surgery or the introduction of rehabilitation exercises so that patients are familiar with what to expect and how to perform these activities early in their postoperative recovery. Similarly, rehabilitation may capitalize on behavior change strategies introduced prior to treatment for long-term maintenance of health behaviors. It is therefore imperative that the two care approaches (rehabilitation and prehabilitation) work in a coordinated fashion before and after treatment to maximize their synergy and respective benefits for patients.

Prehabilitation has also rarely included the breadth of the ‘cancer survivor’ definition, focusing exclusively on the patient, and is not yet inclusive of their caregivers (*i.e.*, family and friends) (16). Many related caregivers of people with cancer experience burnout and caregiver fatigue (17), with levels of psychological distress equal to or often even greater than those seen in the patient (18). The caregiver can experience stress related to disease and treatment cycles that accumulates over time towards an increased risk for illness and psychological morbidity (19), owing to medical (*e.g.*, the unknown regarding diagnosis, prognosis, and clinical course), practical (*e.g*., financial planning), psychosocial (*e.g.*, resolving family conflict) and spiritual/religious uncertainty (20, 21). Unfortunately, supportive care interventions for those affected but not diagnosed with cancer are lacking despite a reduced ability to partake in self-care behaviors (22, 23). A meta-analysis of randomized trials, found that caregivers who receive interventions (including psychoeducation, skills training, and therapeutic counseling) either independently or in conjunction with the patient, experience reduced caregiver burden, distress and anxiety, and improved coping and physical functioning (24, 25). It may be argued that prehabilitation’s benefit for the patient could likely be further enhanced through extension of similar services to caregivers who may be able to support prehabilitation for the patient as well as become more capable of attending to the peri- and post-treatment needs of the patient.

To support evolving clinical and research endeavours in prehabilitation for cancer survivors, we propose a complement to current conceptual frameworks and definitions of prehabilitation (26, 27). The novel contributions of this framework highlight the dynamic and multiphasic potential for prehabilitation that can be applied broadly to the cancer survivor, inclusive of the patient, family, friends and caregivers (16). For the purposes of this paper, we refer to persons receiving cancer treatment(s) as the patient to distinguish them from other cancer survivors. In the sections that follow, we briefly review prehabilitation as a personalized, multimodal intervention, as well as provide an overview of the evidence and theoretical rationale for multiphasic prehabilitation planning, organized by phase of treatment (*i.e.*, neoadjuvant, primary, and adjuvant treatment).

## Multimodal Prehabilitation

While early prehabilitation trials were predominantly unimodal (*e.g.*, exercise or diet alone), contemporary prehabilitation models have adopted a multimodal approach to address the complex needs of people with cancer. Multimodal prehabilitation may be defined as the incorporation of two or more intervention components specifically selected for their potential cumulative or synergistic effects on health outcomes. Multimodal prehabilitation interventions have often comprised a combination of the following: i) aerobic and resistance training to attenuate cardiorespiratory and musculoskeletal deconditioning, respectively; ii) dietary interventions to counteract disease and/or treatment-related malnutrition and to support anabolism and the metabolic cost of exercise; iii) psychological interventions to reduce stress and associated morbidity; iv) cessation of adverse health behaviors (*e.g*., alcohol abuse, smoking); v) medical optimization (*e.g.*, assessing/treating anemia; medication corrections); and vi) behavioral counseling to support intervention initiation and adherence in the pre-treatment setting and establish self-management skills for long-term health behavior maintenance (28–30). While these recommendations are largely driven by expert consensus, recent qualitative findings from patient interviews also support the need for an integrated multimodal approach to prehabilitation (31). These findings are congruent with previous research which suggests that comprehensive prehabilitation support *via* complementary modalities was especially important and well received by people undergoing surgery for lung and colorectal cancer (32).

Inherently, the delivery of multimodal prehabilitation in cancer is expected to incorporate multiple health practitioners that include the oncology physicians (*e.g.*, surgeons, medical oncologists, radiation oncologists, and haematology oncologists) and other medical specialists (*e.g.*, anesthesiologists, geriatricians, physiatrists, and psychiatrists). In addition to physicians, health professionals that direct or deliver specific prehabilitation modalities are also essential. Professions and their respective roles in prehabilitation may include physiotherapists, occupational therapists, kinesiologists, exercise physiologists, dietitians, nutritionists, psychologists, social workers, pharmacists and nurses. To address the needs of the non-patient cancer survivors (*i.e.*, friends and family), health professionals outside of the tertiary care setting may be best suited to prehabilitate for physical or psychological conditioning to support caregiving, bereavement preparation, and/or estate management. Finally, at the heart of person-centered care is engagement of the person with cancer, which represents an essential element of appropriately co-designed interventions and shared decision making. Co-design of prehabilitation interventions by healthcare practitioners and cancer survivors is recommended to cultivate a sense of purpose and responsibility towards managing one’s health *with*, rather than *by*, the healthcare team. Incorporating the patient and caregivers into care planning is aligned with the WHO interprofessional practice definition and supports engagement of cancer survivors towards self-managed behaviors (33).

## Prehabilitation Prior to Surgery and Other Primary Treatments

Despite the breadth of anti-tumor approaches and their distinct consequences to the patient, research on multimodal prehabilitation has almost exclusively focused on surgery. The pre-surgical focus may be explained by the opportunity that wait-times afford to invest in prehabilitation for improvements in peri- and post-treatment health, and potential economic advantages of reduced surgical complications, postoperative morbidity, and length of stay. Addressing modifiable surgical risk factors (such as exercise intolerance, malnutrition, anemia, smoking, and medication usage) have demonstrated a profound effect not only on postoperative HRQoL, but also morbidity, mortality, and the need for further care (34–36). Consequently, surgical prehabilitation has often been thoughtfully tailored to target specific risk factors. For example, surgical prehabilitation commonly includes training to improve cardiorespiratory fitness to prepare the patient for the impending surgical stress response characterized by increased cardiac output and oxygen consumption (37, 38) and because of its established relationship with post-operative morbidity, mortality, and hospital length of stay (39, 40). As a result, cardiorespiratory fitness is often used as a physiological indicator of intervention efficacy.

Systematic reviews of surgical prehabilitation, including both unimodal and multimodal approaches for people with cancer, conclude that prehabilitation improves physical fitness and functional capacity, with lesser, yet still compelling, data to suggest potential improvements in hospital length of stay, post-surgical complication rates, post-operative recovery and HRQoL when compared to usual care or post-operative rehabilitation alone (9–12). The evidence is challenged by limitations in methodological quality, namely small sample sizes, heterogenous interventions and endpoints, and narrow inclusion criteria that limit generalizability. Consequently, prehabilitation has garnered only a weak recommendation for integration into contemporary perioperative care pathways (*e.g.*, Enhanced Recovery After Surgery; ERAS) (41). Moreover, given that many studies fail to appropriately describe safety or adverse events, and higher-risk participants have often been excluded, the actual risk or benefit of prehabilitating frail patients who may need it most is still uncertain. Advancement towards clinical adoption will benefit from ongoing international efforts *via* phase III clinical trials (42–44), as well as improved reporting of safety outcomes, inclusion of higher-risk study populations, well described implementation strategies, and comparisons of multimodal to unimodal strategies that attempt to delineate modality-specific benefit.

Beyond surgery, prehabilitation prior to stem cell transplant (SCT) has received growing research attention given that SCT is a cornerstone haematological cancer management that often follows high-dose chemotherapy or whole-body radiation. The ‘dual hit’ of treatment leaves patients severely deconditioned, where impairment is more apparent in those with poor physical function prior to transplant (45). While interventions delivered after SCT attempt to remediate deconditioning and dysfunction are more widely studied, researchers have also examined prehabilitation exclusively prior to SCT (46–48) or in combination with post-transplant interventions (49–51). Such studies have featured a combination of supervised and self-administered multimodal interventions, comprised of low-to-moderate intensity endurance and resistance training, stress management and relaxation, as well as dietary guidance. The available evidence suggests that prehabilitation for SCT is feasible and may offer favourable changes in physical fitness, psychosocial distress, fatigue, HRQoL and hospital length of stay; however, more research is needed to verify early findings (50). It is worth highlighting that, despite feasibility successes, the research acknowledges significant challenges in delivering prehabilitation prior to SCT in light of the often markedly poor and often changing health status of SCT candidates.

While the surgical and SCT settings currently form the evidence-base for multimodal prehabilitation for primary therapy, comparable preparatory interventions for primary radiation or chemotherapy (among others) remain largely unexplored. It is worth highlighting that the iatrogenic consequences of radiation and chemotherapy may have a more gradual onset than the more abrupt insult of surgery and SCT, and thus the metrics of success may be different across treatments. For example, outcomes of interest in non-surgical contexts, such as chemotherapy or radiotherapy, may prioritize other markers of efficacy, such as dose tolerance, discontinuation of treatment course, and patient-reported health over several weeks of active treatment (*e.g.*, fatigue, cardiovascular function, and psychological health).

## Prehabilitation During or After Neoadjuvant Therapy

Neoadjuvant treatment (NAT; commonly comprising chemo- and/or radio-therapy after surgery, for example) toxicities manifest, in part, as reduced cardiorespiratory and musculoskeletal fitness stemming from underlying tissue, organ, and cellular dysfunction (52–54). Early evidence indicates that this cardiorespiratory deconditioning is associated with an increased risk of surgical complications and peri- and post-operative morbidity and mortality (53, 54). Importantly, cardiorespiratory fitness does not naturally recover between the end of NAT and the time of surgery (55), but rather, continues to decline in the absence of intervention (56). In addition to impaired cardiorespiratory fitness, compromised nutritional status resulting from NAT is common and can worsen physiological dysfunction (57) and affect surgical eligibility (58). Ultimately, NAT creates a more frail, nutritionally compromised surgical candidate that is more likely to have a worse surgical experience. The benefits of prehabilitation in this setting may include the mitigation of NAT-induced deconditioning and consequently promote an earlier and fuller recovery prior to surgery. One practical consideration for prehabilitation in this context is that NAT is often initiated shortly after diagnosis when it may be impractical to routinely intervene prior to its initiation. While initiating prehabilitation prior to NAT may be ideal, there is a growing body of evidence highlighting the health benefits of exercise, enhanced nutrition, and psychology during and after radiation and chemotherapy (59, 60). Collectively, the data suggest that starting prehabilitation during this period with targeted outcomes for both neoadjuvant and primary treatments is likely beneficial.

Interventions aimed at mitigating or preventing associated physiological and psychosocial deconditioning related to NAT have not consistently been described as ‘prehabilitation’, making it difficult to synthesize the relevant literature (61). To our knowledge, exercise delivered concurrently with NAT has been examined in five studies with small samples sizes and variable methodological quality (62–66). Early findings suggest that supervised exercise prehabilitation during NAT is safe, feasible, and may maintain or improve cardiorespiratory fitness over the intervention period. Recently, West and colleagues (56) examined the role of prehabilitation exclusively in the post-NAT/pre-surgical setting in 22 people with rectal cancer who participated in six weeks of facility-based, high-intensity interval training and were compared to 17 usual care participants in a non-randomized trial. Those who participated in prehabilitation recovered cardiorespiratory fitness to baseline levels prior to surgery, whereas usual care participants exhibited suppressed aerobic capacity. These early data highlight the amenability of prehabilitation during this stage of the cancer continuum, given that NAT may be delivered over several months, with a relatively quick and dramatic deconditioning effect, making patients progressively more vulnerable to poor surgical outcomes (52–54). In light of the encouraging early findings, prehabilitation during or after NAT appears to be the most rapidly developing area of the field.

## Prehabilitation Prior to Adjuvant Treatment

**Figure 2:**
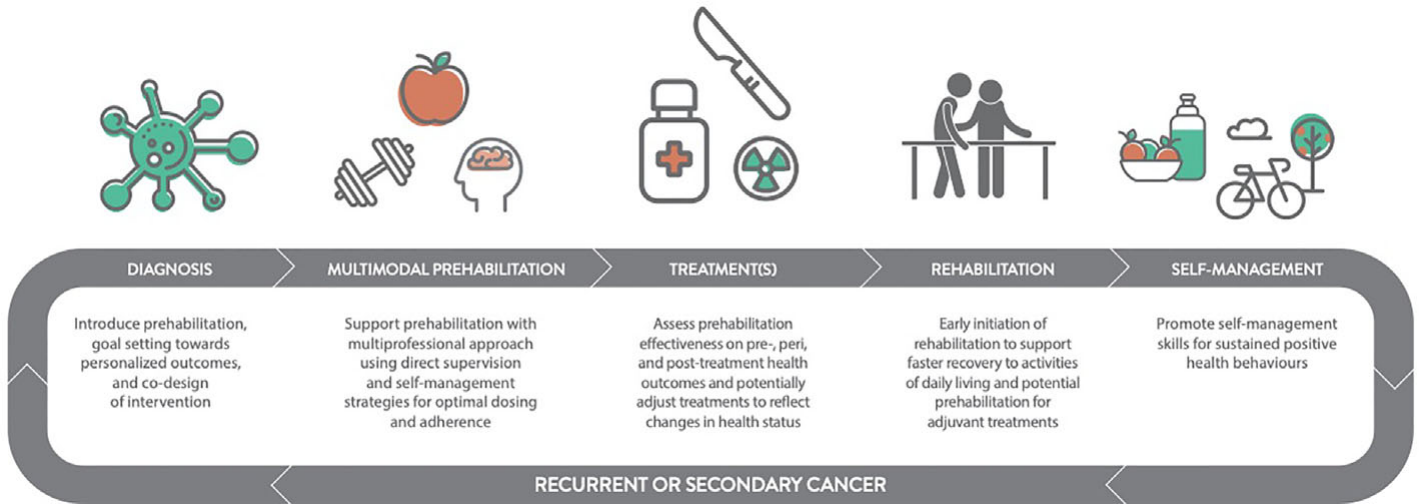
Prehabilitation within the Cancer Continuum (Including Recurrence or New Primary Cancers).

Commencement of early rehabilitation following primary therapy with synchronous or sequential prehabilitation for adjuvant therapy is likely to have both distinct yet complementary functions as shown in **Figure 2**. The initiation of adjuvant therapy is commonly contingent upon recovery and functional status following primary therapy (67, 68), which is important because delayed adjuvant therapy can affect survival (69). It is essential to highlight that re- and prehabilitation in-between primary and adjuvant therapy, are neither mutually exclusive nor synonymous because of their distinctive health objectives. For example, rehabilitation following resective surgery may be required to restore localized mobility and strength, whereas prehabilitation for adjuvant chemotherapy may focus on optimizing cardiorespiratory function to protect against chemotherapy-induced cardiotoxicity. Given that cardiotoxicity can adversely affect tumor control due to reduced dosage amidst concerns of deteriorating cardiac function (70), improving preoperative cardiac resilience appears to be an important strategy as demonstrated in a small, but growing body of pre-clinical research (71–75). Proof-of-concept in humans has recently been demonstrated in a small randomized controlled trial in women with breast cancer, which found that a single bout of vigorous-intensity exercise acutely prior to anthracycline administration attenuated cardiac damage (76). To our knowledge, no studies have specifically examined prehabilitation prior to adjuvant therapy.

Prehabilitation for adjuvant treatment may be particularly beneficial given the compounded deconditioning associated with multiple lines of therapy; and, as a result, these interventions might provide the opportunity to mitigate the catabolic losses and associated consequences of anti-cancer treatments. Martin et al. (77) found that in a cohort of 1,473 people with lung and gastrointestinal cancer exhibiting weight loss, low muscle mass, and low muscle density, survival was just 8.4 months, compared with 28.4 months in patients who had none of these characteristics. Similarly, Prado and colleagues (78) demonstrated that, in patients with metastatic breast cancer receiving capecitabine, the prevalence of dose-limiting chemotherapy-related toxicity in sarcopenic patients was more than twice that of non-sarcopenic patients. Evidence in this setting is limited, but preclinical studies suggest biological plausibility of benefit against chemotherapy-induced cardiotoxicity (71–73); however, human clinical trials are needed for confirmation. In the psychological domain, the deleterious effects of chemotherapy and radiation therapy are well described. In the pre-adjuvant treatment setting, recent findings suggest that approximately one half and one third of patients have anxiety or depression, respectively (79). Importantly, these findings noted the predictive value of demographic factors that warrant consideration for the appropriate tailoring of interventions targeting mental health prior to adjuvant treatment. Studies have also shown that anxiety can be precipitated by concerns regarding physical function and maintaining social roles (80) as well as the financial toxicity of treatment (81), which may be prolonged in long-course adjuvant treatment and could be targets for prehabilitation. There has been little research on psychological prehabilitation prior to adjuvant treatment; however, a recent systematic review and meta-analysis of randomized controlled trials found that “prophylactic” pharmacotherapy, psychotherapy, and other interventions, including exercise, prevented or mitigated depression for those undergoing cancer treatment (82).

## Multiphasic Prehabilitation: A Conceptual Framework

**Figure 3:**
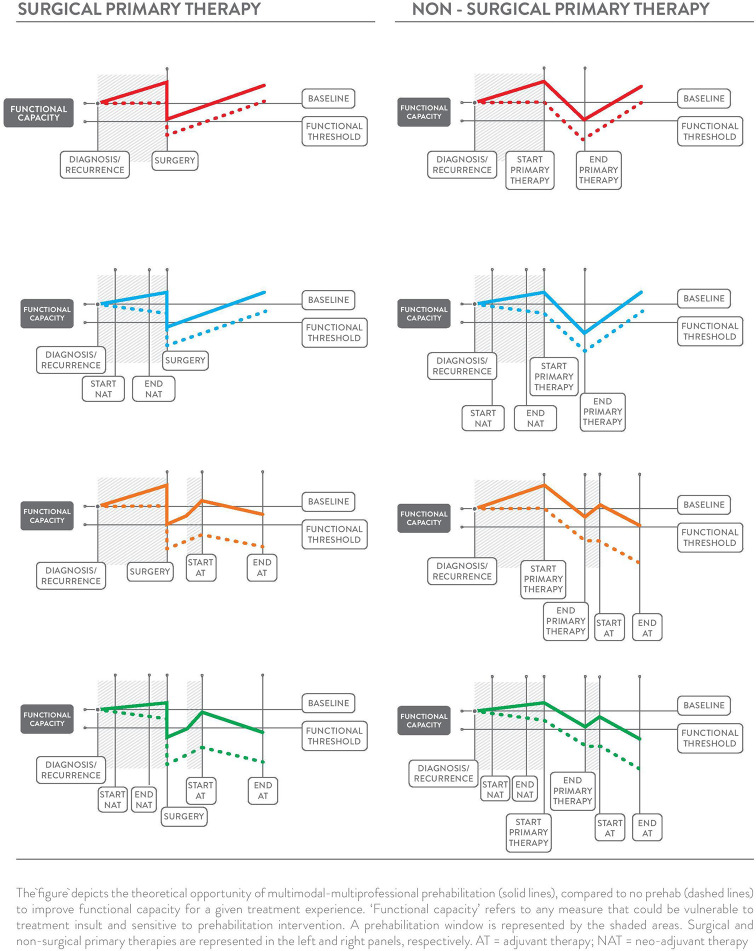
Prehabilitation Across the Cancer Continuum.

Multiphasic prehabilitation, as a novel and complementary conceptual framework for the field, is depicted in the panels of **Figure 3**. It incorporates and extends early and revised models of prehabilitation described by Carli and colleagues (26, 27) and the cancer-specific definition by Silver, Baima, and Mayer (83) to provide an evidence and theory-informed application of prehabilitation across the entire cancer continuum. This framework is intended to guide future research by connecting the burgeoning data that show the benefit of healthier cancer survivors prior to different treatments and combinations of treatments with the body of evidence on modifiable risk factors for adverse treatment- and health-related outcomes. Core to the multiphasic concept is that prehabilitation may be considered as a health optimizing strategy that can occur multiple times following an initial cancer diagnosis. Multiphasic prehabilitation is an innovation to initial conceptualizations that has yet to be empirically tested as a cohesive sequence of preparatory measures across treatment exposures. Nevertheless, it is intended to provoke investigation of proactive interventions that focus on periods of relative health where the ‘maximum tolerable dose’ for a health intervention can be pursued more readily in the absence of active treatments that often erode functional capacity, appetite, mental health and motivation. Multiphasic prehabilitation requires nuance and tailoring to the existing and anticipated experiences at each phase of the cancer journey to minimize treatment-related side effects and subsequent treatment delays, thereby improving wellbeing and potentially prognosis over the long term. Aggressively preparing for repeated challenges across the trajectory of survivorship with multiphasic prehabilitation may be akin to periodization training models of high-performance sport with cyclic rounds of training prior to competition, both with similar goals: to optimize health preceding an anticipated stressor to ensure ‘maximal performance’ and rapid recovery.

## Future Directions in Prehabilitation Research

The efficacy for prehabilitation on health and economic outcomes has been best demonstrated in the surgical setting; however, limitations in methodological quality must be addressed to compel widespread adoption into perioperative care. Emerging areas of prehabilitation in oncology, including prehabilitation prior to non-surgical anti-tumor treatments have shown promising findings and justify further examination, including within the context of a multiphasic approach. As the volume and quality of evidence describing the benefits of prehabilitation mounts, important information about its delivery in a clinical setting is needed. Methodologies that assess complex interventions, such as process evaluations as highlighted by the Medical Research Council (84) will permit greater understanding of biological, psychological, social and behavioral (‘biopsychosociobehavioral’) factors that drive prehabilitation participation, adherence, and medical outcomes in complex healthcare settings. Similarly, implementation science methodologies, as well as research within the context of clinically integrated programs, will add rich evidence to the understanding of how prehabilitation can be incorporated into standard of care as well as impacts on patient and economic outcomes. Examples of prehabilitation programming are occurring worldwide, including initiatives in Australia (85), Canada (32), Denmark (86), Japan (87), the Netherlands (88), Spain (89), the United Kingdom (90), and the United States (91, 92). Finally, across all research designs and settings, important gaps in research include: i) a better understanding of the differences between unimodal and multimodal prehabilitation and for which cancer survivors these should be applied; ii) strategies to identify and adapt prehabilitation for ‘non-responders’; iii) prehabilitation for non-patient cancer survivors whom are likely to experience significant decline in aspects of their health when supporting a patient; and iv) the mechanisms of benefit of prehabilitation for cancer survivors.

## Conclusion

The concept of prehabilitation has rapidly ascended into the common lexicon of survivorship care with research across cancer types, treatments, and modalities. The proposed conceptual framework for prehabilitation aims to guide further investigation of the viability and impact of repeated, pre-treatment interventions that target improved health outcomes throughout the entire cancer continuum.

## Author Contributions

All authors listed have made a substantial, direct, and intellectual contribution to the work and approved it for publication.

## Conflict of Interest

The authors declare that the research was conducted in the absence of any commercial or financial relationships that could be construed as a potential conflict of interest.
